# Identifying mechanisms of youth mental health promotion: A realist evaluation of the Agenda Gap programme

**DOI:** 10.1371/journal.pmen.0000068

**Published:** 2024-06-18

**Authors:** Justin Jagosh, Corey McAuliffe, Liza McGuinness, Trevor Goodyear, Rebecca Haines-Saah, Zachary Daly, Tanya Halsall, Taylor G. Hill, Tyesa Kruz, Joan Wandolo, Tasneet Suri, Emily Jenkins

**Affiliations:** 1 Centre for Advancement in Realist Evaluation and Synthesis (CARES), Vancouver, Canada; 2 School of Nursing, University of British Columbia, Vancouver, British Columbia, Canada; 3 Wellstream: The Canadian Centre for Innovation in Child and Youth Mental Health and Substance Use, Vancouver, Canada; 4 Cumming School of Medicine, University of Calgary, Calgary, Alberta, Canada; 5 Institute of Mental Health, University of Ottawa, Ottawa, Ontario, Canada; 6 Department of Psychology and Neuroscience, Dalhousie University, Halifax, Nova Scotia, Canada; 7 Agenda Gap youth participant, Richmond, British Columbia, Canada; Teesside University, UNITED KINGDOM

## Abstract

Promoting youth mental health is a critical public health priority and merits robust policy and practice responses, inclusive of youth-centred and upstream interventions that address the root factors contributing to mental health outcomes. To that end, non-familial youth-adult relationships can powerfully impact youths’ healthy development, mental health and well-being, and capacities for enacting change within home, school, and policy contexts. Agenda Gap is a youth mental health promotion programme based on this principle, in which adult facilitators support youth (aged 15–24) in co-exploring activities focused on mental health promotion and policy advocacy, while fostering supportive, trusting intergenerational relationships. This study presents a realist evaluation of Agenda Gap, drawing on realist qualitative interviews with youth participants (n = 18) and adult collaborators (n = 4). We constructed 15 initial programme theories in four theoretical areas that we subsequently explored via analysis of the realist interview data. The analysis used the middle-range theories of ‘Third Space’ and ‘Third Place’ to conceptualize and articulate how causal mechanisms were produced from the non-familial youth-adult relationships that are foundational to the programme. Results are presented across 10 context-mechanism-outcome (CMO) configurations in three sections: (1) Agenda Gap Facilitation Activates ‘Third-Space/Third-Place’ Mechanisms; (2) Youth Enhance Personal Mental Health while also Supporting Mental Health of Friends and Family; (3) Youth Become Inspired and Informed to Act as Advocates and Policy Change Agents. Taken together, these findings provide insights into the important mechanisms of non-familial intergenerational trust building and demonstrate how a strengths-based conceptualization of youth mental health supports mental health maintenance, promotion, and advocacy for this population.

## Introduction

Youth worldwide face growing struggles with their mental health due to complex environmental, social, and technological transformations and associated stressors [1]. Indeed, since the onset of the global COVID-19 pandemic in early 2020, there have been significant increases in mental health challenges among children and youth [2]. Given this, and evidence indicating that most mental health conditions first arise during childhood and adolescence, the promotion of mental health in youth contexts is a critical public health priority [3]. Through its strengths-based orientation, mental health promotion advances positive mental health and equity by building on the capacities of individuals and their communities to respond to upstream determinants, or root factors, shaping mental health and illness [4]. Specifically, mental health promotion is designed to address the social and structural determinants of mental health, such as the presence of social supports, safe and healthy environments, and access to education and meaningful employment, that influence health and well-being across socioecological levels–that is, for individuals, families, communities, and whole populations [3–7].

In youth settings, mental health promotion programming has been shown to support and enhance healthy development and well-being with various benefits. For example, research has documented growth in leadership skills and health promoting behaviours, feelings of empowerment, sense of responsibility and problem solving, and self-confidence [8–11]. Beyond the individual level, mental health promotion programming for youth has been found to strengthen relationships within peer and family networks, shift community norms and practices, and change social conditions to reduce health and social inequities [5, 12, 13].

The theory of positive youth development, which focuses on the protective factors that help young people to achieve developmental tasks and flourish [14] suggests that the benefits associated with mental health promotion programming may reflect, in part, the non-familial youth-adult relationships that are central to programme implementation [15]. In exploring the characteristics of positive non-familial youth-adult relationships, the importance of authentic, consistent, and enduring relationships have been identified as critical [16–18]. Within these relationships, a sense of connectedness, inclusive of feelings of closeness and belonging between youth and caring adults, such as teachers and mentors, greatly benefits and facilitates positive outcomes [10, 15, 18–22]. The opportunity to engage in supportive relationships with adults encourages youth to be active participants in their own development [10, 18, 23]. Importantly, research has identified that “engaging and transformative youth-adult relationships exert the greatest impact on youth who are the most marginalized” [24]. Indeed, although these relationships often have some degree of benefit for all youth, there is evidence of larger developmental effects among youth with relatively fewer resources [19, 24].

While the health and developmental benefits of non-familial youth-adult relationships have been widely studied, the mechanisms through which these relationships operate to produce positive outcomes have not been well articulated in the mental health promotion literature. This realist evaluation–grounded in data from Agenda Gap, a youth mental health promotion programme that equips youth to lead systems change through policy advocacy–contributes to addressing this gap by building applied evidence to guide responsive and equity-oriented mental health promotion practice with youth. Specifically, this paper explores the causal mechanisms underpinning impactful adult mentorship and facilitation within the context of youth mental health promotion programming. Such empirical insights are needed alongside theoretical developments to advance mental health promotion frameworks and practice.

### Intervention overview

Agenda Gap is a Canadian youth mental health promotion programme designed to build skills and capacities among youth aged 15–24 years to engage in policy advocacy to strengthen positive mental health across socioecological levels. Cohorts of youth and adult facilitators connect for approximately 48 hours of programming–drawing on various modalities to build knowledge, skills, and applied experience–over a 6-month period. Youth are recruited through community-based partners who host the group. To date, these partners have included community- and youth-serving organizations as well as school districts. Recruitment strategies are tailored by site, but involve a combination of recruitment posters and social media posts, as well as programme information sharing by school and community-based staff. Interested youth reach out to the research team to coordinate an interview to assess interest and fit. Facilitators include members of the Agenda Gap team and those identified from within the community, with the intention of building local capacity for ongoing programme implementation. Facilitators bring experience in working with youth and receive training from the Agenda Gap team in trauma- and violence-informed practice, principles of meaningful youth engagement, and mental health promotion. Facilitators provide support and engage youth in programme activities aimed at enhancing knowledge and applied skills in mental health promotion and policy advocacy, through fostering healthy and supportive non-familial youth-adult relationships. In addition, the programme includes activities that link youth with external adult allies working in school, community, and policy arenas to construct bridges and create conditions wherein youth experience benefits of their advocacy efforts. Agenda Gap has been delivered as a virtual, hybrid, and face-to-face programme. More details regarding the history of Agenda Gap and the core programme content have been published elsewhere [25]. Outcomes of interest include benefits at the individual level (e.g., self-efficacy, strengthened positive mental health), interpersonal and familial levels (e.g., improved ability to support or share mental health knowledge with family/peer groups), and community and societal levels (e.g., youth-centred policy and systems change in school and community contexts) (see Jenkins et al. [5] for overview of programme outcomes).

## Methods

### Study approach

Realist evaluation [26] was used to investigate how Agenda Gap works, for whom, and under what circumstances (see S1 Appendix for definition of terms pertaining to realist evaluation methodology). Realist evaluation illuminates context-mechanism-outcome (CMO) configurations to unpack causal mechanisms of complex interventions and how these are triggered by or within key elements of the context. Initial programme theories (IPTs) are constructed from literature reviews and key informant interviews with programme architects or implementers to produce hypothetical explanatory insights into the nature of intervention components and how they function to produce intended and unintended outcomes [27]. The process of building IPTs helps research teams clarify the programmatic architecture in question and shapes the study design. Substantive middle-range theories relevant to the causal propositions are used to scaffold theoretical insights and provide higher-level conceptualizations of causal pathways [28]. Data, including that collected through realist qualitative interviews, is then collected to test IPTs [29]. Finally, the logic of the CMO configurations is used to assemble and present generative causal claims supported by the data [4].

IPTs for Agenda Gap were formulated by the research and programme design team through an iterative process involving discussion and review of scientific literature in the areas of youth citizenship, liberation psychology, youth development, and mental health promotion. A total of 15 IPTs spanning four theoretical areas were initially developed to articulate the architecture of the Agenda Gap programme (see Table 1). These include: (1) screening, selection, and programme preparation; (2) intervention content and approaches; (3) programme facilitation; and (4) youth-adult ally linkage and collaboration. The IPTs were then employed to generate an interview guide (see S2 Appendix) used to conduct realist qualitative interviews that could further test and refine the IPTs [29]. Substantive middle-range theories relevant to the causal propositions were sought to scaffold theoretical insights and provide higher-level conceptualizations of causal pathways [28]. Finally, the logic of the CMO configurations was used to assemble and present generative causal claims supported by the data [4].

**Table 1:** 15 initial programme theories across four theory areas of the Agenda Gap architecture.

**IPT AREA #1. SCREENING, SELECTION AND PROGRAMME PREPARATION** **1.1: Identification of potential participants by programme hosts.** Community organizations who ‘host’ the programme will play a key role in identifying and recruiting youth who may be interested in participating due to history of advocacy work or lived experience of health and social inequities. **1.2: Selection process:** The screening and selection process (involving an initial one-to-one interview with youth candidates for the programme) ensures that the youth who join have common or shared experiences. Such group curation will promote positive group dynamics and allow the identification of collective priorities for policy and systems change. **1.3: Readiness:** The screening and selection process ensures that youth who enter the programme have an adequate level of commitment and understand their roles and responsibilities in the programme. The screening process leads to lower attrition and increased participation and retention. **1.4: Monetary incentivization**: Monetary incentives (payments made to youth to compensate for their time participating in the programme) reduces barriers to participation in terms of competing priorities, including other employment. Monetary incentives also convey that youth expertise are valued leading to feelings of validation and legitimacy. This is especially impactful for youth whose voices have historically been absent from policy and other decision-making. **IPT AREA #2: INTERVENTION CONTENT AND APPROACHES** **2.1: Mental health promotion orientation:** Youth participants will acquire an awareness of the social and structural determinants of mental health through information and discussion provided in the programme. This will empower an appreciation for the collective (not exclusively individual) responsibility for protecting and strengthening youth mental health. This awareness will allow youth to recognize gaps in mental health-relevant policies and feel motivation and capacity to advocate for change. **2.2: Multimodal content:** The multimodal programme content (e.g., video, audio, role-play, reflection, discussion, application) supports the diversity of learning styles among the youth, increasing critical thinking, interest, retention, and motivation. **2.3: Interactive format:** The interactive format in which youth collectively work through the programme content creates active, experiential learning, and intensive dialogue. This leads to increased social connectedness, strengthened sense of agency, and improved mental health literacy. **2.4: Duration and timing of sessions:** Duration and timing of the sessions (too long or too short) has an impact on youth interest and retention. A minimum timeframe and dose is needed to adequately build rapport and trust to sustain engagement and positive impact. **IPT AREA #3: PROGRAMME FACILITATION** **3.1: Creating psychological safety:** Adult facilitators ensure psychological safety in the group by modelling healthy communication and using a strength-based approach to support youth in identifying their assets. Adult facilitators assess and refer to other supports when personal issues arise during workshop activities and interaction. **3.2: Managing youths’ expectations:** Adult facilitators will help to bring emergent ideas from youth participants about mental health advocacy and policy change into a realistic frame, which will allow youth to maintain enthusiasm, identify successes, and reduce frustration or sense of disappointment. **3.3: Fostering intergenerational trust building:** Youth who do not have strong existing connections with adults have the opportunity to develop relationships with the adult facilitators. This experience builds a strong sense of trust in adults, which can improve relationships with other adults, including those with whom they engage for policy change outside the Agenda Gap programme. **IPT AREA #4: YOUTH–ADULT ALLY LINKAGE AND COLLABORATION** **4.1: Liaising with external community supports:** Host organizations and adult facilitators draw on a network of community linkages and promote youth collaboration with other adults in organizational/policy arena. This leads to effective policy engagement and relationship building between youth and adults. Having practice engaging with adult facilitators, youth will feel increased confidence in communicating with other adults in their policy advocacy efforts. **4.2: Policy makers realize youth assets:** Policy makers are engaged through the programme and have an opportunity to listen and collaborate with youth. They realize the capacity that youth have for making meaningful contributions to policy, leading to efforts to shift practices to ensure youth are provided opportunities to contribute to policy decision making on an ongoing basis. **4.3: Youth appreciate persistent inequities:** Youth collaborating with policy makers will appreciate the power disparities in society that make advocacy work difficult. They may feel discouraged by the experience of collaborating with policy makers in the real world, reducing motivation to engage in advocacy work in the future. This experience may also put a strain on their mental health. **4.4: Youth supported despite unresponsive or pessimistic policy makers:** When youth interact with policy makers who are unresponsive or pessimistic in relation to their ideas for change, they may feel frustrated. However, if they are provided with strategies for coping with frustration and disappointment in the programme, they may be better positioned to continue and sustain their engagement and motivation to incite change.

### Data collection

A total of 18 youth aged 15–17 years were recruited between September 8, 2020 and December 7, 2020 to participate in one of two virtual Agenda Gap cohorts implemented in British Columbia, Canada, between fall 2020 and spring 2021. All 18 of these youth participated in a realist qualitative interview within 1-month of completing their respective Agenda Gap programme (i.e., between June 2021 and November 2021). Four of the 10 adult allies who supported these Agenda Gap cohorts also participated in an interview, the remainder were unavailable during the data collection period. Realist interviews were conducted online (via Zoom) by three female members of the research team (CM, LM, EJ) who were familiar to the participants and experienced in qualitative data collection. All interviews lasted between 60–90 minutes and were audio recorded, transcribed, and accuracy checked. Transcripts were labelled “Y” for youth or “AS” for adult supporter and assigned a code number. Quotations referenced during analysis were also tracked by transcript line number.

### Ethics statement

Ethical approval for the study was obtained by the University of British Columbia Behavioral Research Ethics Board (H17-001602). Informed consent was provided by all participants verbally prior to the beginning of each interview. Written informed consent from the youth participants’ legal guardian/next of kin was not required to participate in this study, in accordance with national legislation and institutional requirements regarding young peoples’ capacity to consent to participate in research. Youth participants received a $20 honorarium to acknowledge their time and contributions, while adult participants contributed to interviews as part of their professional role.

### Data analysis

All interview data were uploaded to NVivo 12 to support data management. The realist analysis was led by JJ, who brings extensive experience in realist evaluation, including as the Director of the Centre for Advancement in Realist Evaluation and Synthesis (CARES). JJ read and coded all the transcripts by searching for causal insights that had relevance to the IPTs. The first five transcripts were read non-analytically to achieve a general understanding of the content and quality of the data. Transcripts were then re-read by JJ, CM, LM, and EJ to code passages containing important causal insights into the Agenda Gap programme that aligned, disputed, or extended original IPTs and held important implications for the programme. A guiding principle of data extraction was pragmatism, as espoused in realist evaluation, to prioritize data that are important for improving the functioning of the programme and tailoring to diverse contexts and population needs [30]. Data were then assembled by JJ into a mini analysis involving CMO configurations organized across subheadings mapped onto the formal and informal Agenda Gap programme architecture.

As a base framework for further data extraction, subsequent transcripts were analyzed to highlight and extract additional key passages detailing causal insights into how Agenda Gap works, for whom, and to what extent. Transcript passages were incorporated on an on-going basis. As data were included in the analysis, headings evolved and CMO configurations were revised. The resulting CMO configurations represent nuanced re-articulations of the causal insights offered by interviewees. They convey causal claims, with reasonable extrapolations made to extend the mechanism to outcomes and add context where such articulations were not made explicit.

#### Middle-Range theory: Third Space and Third Place theories

**Fig 1:**
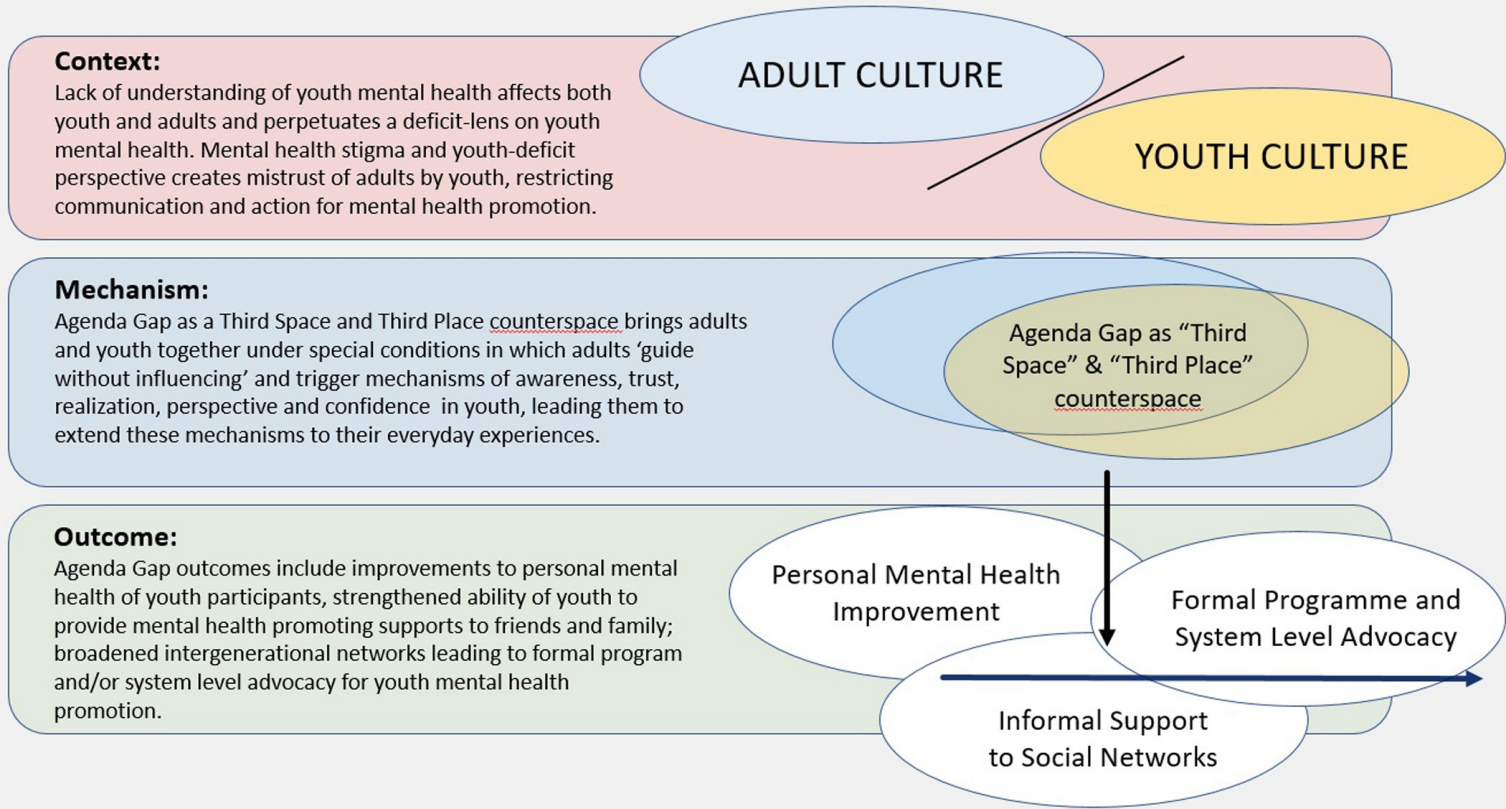
Using theories of Third Space and Third Place counterspaces as a conceptual scheme for data analysis.

Through data immersion and engagement with realist evaluation literature, the research team identified that the middle-range theories of ‘Third Space’ and ‘Third Place’ served to support CMO configurations constructed through analysis. Third Space Theory conceptualizes the value of co-created social spaces, which are separate from normative spaces such as home and school [31]. As noted by Soja [32], “First and second spaces are two different, and possibly conflicting, spatial groupings where people interact physically and socially: such as home (everyday knowledge) and school (academic knowledge). Third spaces are the in-between, hybrid spaces, where the first and second spaces work together to generate a new third space”. Third Place Theory further extends this notion with implications for youth who experience marginalization. For example, Littman [33] suggests that such youth benefit from settings beyond home and school where they can learn adaptive responses–rooted in community and social capital–to navigate injustice and discrimination while maintaining well-being. These two theories hold relevance to youth mental health promotion programming, and Agenda Gap specifically, in that they direct attention to the need for special ‘in-between’ social or counterspaces that inspire youth and strengthen their resources for mental health. Counterspaces are contexts where deficit-oriented dominant cultural narratives are proactively avoided and challenged [34]. Such spaces are particularly relevant for youth, who are already negotiating their identities as they transition from childhood to adulthood and can feel disempowered in the normative spaces of school and home. Informed by this theoretical framing, Agenda Gap can be conceptualized as a protective and distinct intergenerational counterspace that creates an environment for mutual respect, growth, and adaptive coping for participants. Fig 1 depicts the use of these theoretical ideas to scaffold the CMO configurations.

## Findings

Of the 18 youth participants, the majority self-identified as girls/women (n = 17), while one identified as a boy/man. These youth reflected diversity in ethno-racial background, with the majority identifying as Southeast Asian (n = 8), followed by East Asian (n = 6), Middle Eastern (n-2), Black (n = 1) and mixed white and Indigenous descent (n = 1). Of the adult ally participants, all four were women and contributed to Agenda Gap through their professional positions in the health (n = 1) and education (n = 3) sectors.

The study findings are organized in three sections: Section A reports on the inner workings of the programme and maps CMO configurations in relation to the experiences of youth participants during Agenda Gap sessions. Section B reports on the CMO configurations that describe how the experiences of being a youth participant in Agenda Gap affected personal mental health as well as the inclination to provide knowledge and informal support to peers and family. Section C reports CMO configurations on how Agenda Gap helped youth gain perspective and practice to engage formally in efforts to create system-level change addressing social and structural determinants of mental health. Table 2 summarizes these findings.

**Table 2:** CMO configurations thematically organized into three sections.

**Section A: Agenda Gap Facilitation Activates ‘Third-Space/Third-Place’ Mechanisms:** CMO #1: Facilitators break intergenerational hierarchy CMO #2: Facilitators express democratic, affiliative leadership by *guiding* not *directing* group activities with youth CMO #3: Facilitators encourage youth to share their perspectives while also creating a “no pressure” environment CMO #4: Facilitators balance serious discussions about mental health and illness with small-talk, levity and humour **Section B: Youth Enhance Personal Mental Health while also Supporting Mental Health of Friends and Family:** CMO #5: Youth learn tools and techniques to strengthen personal mental health CMO #6: Youth understand mental health as a positive resource CMO #7: Youth appreciate how to use mental health knowledge to support friends and family **Section C: Youth Become Inspired and Informed to Act as Advocates and Policy Change Agents:** CMO #8: Youth identify policy gaps and initiate actions intended to create systems-level change CMO #9: Youth become skilled in different approaches to advocacy and incorporate thoughtful, balanced responses to support action CMO #10: Youth gain awareness of adult ally perspectives on youth mental health promotion and experience expanded networks and strengthened relationships with non-familial adults

### Section A: Agenda Gap facilitation activates ‘Third-Space/Third-Place’ mechanisms

The data demonstrated that programme facilitation requires a specialized approach. When this is accomplished, it creates intergenerational ‘third space’ and/or ‘third place’ conditions, which are counter to what youth typically experience when interacting with adults. Youth shared that adults in their everyday lives (i.e., parents, teachers) can be judgemental and quick to problem solve, rather than allowing youth to express themselves and come to their own solutions. In Agenda Gap, youth participated in a different dynamic with adults, stimulating their interest, motivation, and confidence. Section A presents details of how facilitators created this third space/third place counterspace.

#### CMO #1: Facilitators break intergenerational hierarchy

The data demonstrated that the sensitivity required for youth to feel safe is supported by facilitators breaking with intergenerational hierarchy. The adult facilitator role in Agenda Gap was critical in fostering a safe space wherein youth feel validated. Facilitators created a connection with youth that was different from their everyday interactions. This was achieved through facilitators sharing personal experiences without oversharing. The contrast between the way adult facilitators engaged with youth and what youth are accustomed to triggered mechanisms of motivation in youth to engage.

*[Agenda Gap] wasn’t so much institutionalized*, *like [adult facilitators saying] “I’m an adult*, *I’m a part of this and I’m using you for information”*. *It was more*, *“I want to hear your story*. *I want to hear what you’ve been through*. *I want to learn from you”*. *That is not something I’ve ever experienced with an adult ally before*. *[6Y*, *71]*

Rapport, relatability and bonding between adult facilitators and youth participants was important to the co-creation of a safe space to engage in the programme. For facilitators, the effort to build trusting relationships with youth meant finding a balance between breaking with intergenerational hierarchy, while at the same time, not acting too casual and obscuring the importance of being an adult role model.

*[Adult facilitators] would talk about their own endeavors and what makes them happy and things they’re proud of*, *not so proud of*, *and instead of putting on this corporate front of like*, *“I’m your facilitator*, *I’m perfect”*. *It was very humanized*. *Um*, *and you kind of forgot that you needed a facilitator at all because the conversation just flowed after that*. *[6Y*, *318]*

While the importance of creating personal connection between youth and adults featured strongly in the data from youth participants, an adult ally emphasized the importance of finding a balance between sharing stories to bond with youth, while not over-sharing–as it risks being counter-productive or even harmful.

*Sometimes [adults sharing stories of their experience as a youth] can be a bonding thing*, *but always coming back to the question: is this going to be helpful and appropriate for this youth in this situation?* …*Sometimes adults [are] like*, *“oh yeah*, *I remember when I used to get really drunk when I was a teen”*. *And it’s like*, *is that what’s needed in this moment? [12AS*, *103]*

*Context*. Youth are habituated to relationships with adults looking down upon them, thereby limiting the safe space in which they can express mental health needs or challenges.

*Mechanism*. Adult facilitators share personal stories (resource) that allow youth to see their humanity (response) and increase a feeling of relatability (response) and equity within the group dynamic. Youth feel safe to participate (response). By sharing, but not oversharing (resource), adults maintain their roles and youth feel safe to explore their feelings and ideas (response).

*Outcome*. Youth increase their engagement in the programme with benefits to their personal mental health and advocacy work.

#### CMO #2: Facilitators express democratic, affiliative leadership by *guiding* not *directing* group activities with youth

Youth described the positive impact of having facilitators role model being open-minded and accepting of divergent points of view, while also guiding the group without intentionally directing their interests or perspectives. Youth emphasized how the language of the facilitators set the tone for the discussions and how they were able to role model these attributes.

*One of the participants said something that I personally disagreed with*, *and I was ready to go off*. *But she [facilitator] did say that like*, *you know*, *“I disagree”*, *but at the same time she didn’t yell at the participant or anything*. *She like took it calmly and tried to understand their perspective and tried to be accepting to them as well so that everyone feels included*. *And seeing that made me feel better*, *too*. *[4Y*, *524]*

The balance required in navigating guidance alongside an openness to different perspectives was important because it supported youth to continue to explore and experiment with ideas within the boundaries of a safe environment. These experiences further allowed youth to consider that different ideas and ways of thinking may deepen their own understandings of mental health.

*There was no negative bias towards anybody for what they said*. *‘Cause if I said something and then my colleagues said “no*, *I don’t think so”*, *there wouldn’t be any animosity or*, *“Oh*, *so I can’t work with you”*. *It’s not like that*. *It was more*, *you know*, *“thank you for giving me another layer to my insight…I appreciate you bringing that up”*. *So their language is very accepting*. *[6Y*, *551]*

*Context*. Youth can experience many forms of oppression and are in a developmental stage where they are exploring their evolving views and ideas. At times, this can create fear of saying the wrong thing or being ostracized for expressing certain perspectives or experimenting with ideas.

*Mechanism*. Facilitators role model how to be accepting of divergent views by communicating honestly. When they disagree, they maintain openness to hearing and understanding different viewpoints (resource). This inspired youth participants to express themselves authentically, knowing they are safe to explore their ideas and practice being open-minded to others’ views (response).

*Outcome*. Improved group cohesion and safety; youth having strengthened capacity to manage diversity in their everyday conversations from exposure to the role modelling of the facilitators.

#### CMO #3: Facilitators encourage youth to share their perspectives while also creating a “no pressure” environment

Youth participants described the importance of facilitators creating a “no pressure” environment wherein youth could participate in ways that felt comfortable. This contributed to a relaxed environment that evoked authentic engagement. Finding a balance between a “no pressure” approach and encouraging youth to participate was also important; however, youth suggested that when facilitators remove pressure, it is tempting to become a passive listener in the group.

*It’s important to respect our choices*, *but sometimes it’s also helpful to push*, *to push ourselves to our limit… If it’s up to our choices*, *it’s easy to fall into the trap of always not choosing to answer*. *But maybe make it like a conversation*, *not like*, *“oh this a serious conversation that you need to say something about”* … *Just make it like a conversation*. *And I think the facilitators are already doing a good job about that*. *[5Y*, *541]*

Youth described the benefit of having moments of anonymous participation (e.g., using anonymous functions in the virtual environment to express feelings), and how this, in turn, stimulated their active participation. Youth appreciated how facilitators found a balance in encouraging engagement without judgement about how or what participation should look like.

*I think that*, *especially when it comes to the topic of mental health*, *they were able to kind of like take a step back and be like*, *okay*, *“if you feel comfortable… really appreciate if you would contribute*, *but if not*, *don’t worry about it*.*” [10Y*, *292]*

*Context*. Talking about mental health and illness and the various contributing factors may cause discomfort for some. Youth may tend toward remaining silent during discussions if their participation is not encouraged.

*Mechanism*. Facilitators strike a balance in helping youth to feel comfortable in their participation (anonymous contribution, openly contributing perspectives, etc.) (resource) and communicating the various ways that youth can participate (resource), leading youth to feel safe in discussing sensitive or complex topics (response).

*Outcome*. Youth actively participate in the programme and overcome a reluctance to contribute to discussions related to mental health and illness; enhanced confidence.

#### CMO #4: Facilitators balance serious discussions about mental health and illness with small-talk, levity, and humour

Youth participants also described how facilitators struck a balance between engaging youth in serious discussions about mental health and illness while maintaining a sense of humour and levity. That balance created safety in the context of these conversations. Activities underpinned by principles of social and emotional learning were utilized to connect with youth’s feelings and current experiences in a way that offered lightheartedness and ease. Furthermore, youth described these activities as creating an uplifting atmosphere, wherein they felt comfortable and safe discussing serious topics such as mental health challenges and social injustices.

*They really built in an environment that would uplift your mood…I think it was extremely positive and understanding and welcoming to any sort of mental state*. *We have an icebreaker question at the beginning of each session and a lot of the time it would be like*, *“if you had to rate your week with a fruit*, *what would it be?” …And I was very open to that by the end*, *everyone was feeling pretty good*, *no matter how they started*. *[8Y*, *292]*.

*Context*. Conversations about mental health and illness can be difficult to have and youth may feel reluctant to participate.

*Mechanism*. Facilitators strike a balance between the focus on serious topics and providing lightness, levity, and humour (resource), leading youth to feel they are in a safe space that can be enjoyable and productive (response).

*Outcome*. Social cohesion in the group; improved youth mental health; efforts toward mental health advocacy and change.

### Section B: Youth enhance personal mental health while also supporting mental health of friends and family

Youth participants reported that Agenda Gap positively impacted their mental health. They attributed this to the programme’s strengths-based orientation, the friendship and respect offered by facilitators, and in-session social and emotional learning activities to build skills for well-being. Section B describes the tools for well-being that youth acquired through their participation in the Agenda Gap programme. It further articulates how these skills helped youth participants to cope with everyday stressors and to improve their capacity to support people close to them, including friends and family.

#### CMO #5: Youth learn tools and techniques to strengthen personal mental health

Agenda Gap participants acquired skills to maintain and improve their personal mental health. This included learning and applying social and emotional learning techniques (e.g., meditation, emotional awareness exercises and reflective writing), shown to decrease challenging emotional states, including stress and overwhelm. The maintenance of personal mental health was seen as necessary for learning how to be a positive force in systems-level change.

*I’m also not only learning about policy and how things work*, *but I’m also learning how to do things in my daily life to have better mental health*. *Like we would often do breathing exercises or meditation… the ending [of the session] would always be things that we can do in our daily lives…because I’m obviously learning all these educational things*, *but I’m also learning applicable things that are good for my life*. *[4Y*, *311]*

*Context*. Youth commonly experience stress and overwhelm, which can limit their confidence and well-being. Such challenges can be supported by accessible techniques that can help to better manage emotions and self-regulate.

*Mechanism*. Facilitators introduced social and emotional learning techniques (resource), leading youth to realize that these tools can provide important supports for their mental health (response).

*Outcome*. Improved mental health and ability to support others; improved capacity and adaptive coping for advocacy work.

#### CMO #6: Youth understand mental health as a positive resource

Through participation in the Agenda Gap programme, youth expanded their understanding of mental health, including the ability to distinguish between mental health and mental illness. For some, this involved a shift from deficit- to strengths-based views of mental health.

*So I think the fact that they defined it [mental health] as mental wellness made me feel like maybe everyone goes through this…That actually had a really good effect on me mentally*, *too*, *you know mental health getting defined as something else than just mental illness*. *[12Y-188]*

Youth also gained perspective on how to handle mental health ‘ups and downs’, realizing that some measure of acceptance of challenging feelings is part of growing and coping in the face of mental health adversity.

*One thing I feel was very stressed in the meetings was that it’s okay to not be okay*. *Like it’s okay to not always be happy*, *like be smiling and everything* …*this is how I’ve learned that this is okay*, *and that I don’t have to be fine all of the time*. *[7Y*, *555]*

*Context*. Societal norms about mental health and illness contribute to a culture of silence, whereby people feel pressure to act as if they are fine, even when they are not coping well. Such pressure can exacerbate mental health challenges due to a lack of outlets to express oneself and share difficulties.

*Mechanism*. Agenda Gap facilitators introduced a strengths-based orientation to mental health (resource) in which youth came to the perspective that ‘it’s okay to not be okay’ (resource). These efforts led youth to feelings of safety and a motivation to explore their individual and social challenges without self-judgement (response).

*Outcome*. Improved mental health; improved coping and willingness to explore range of emotional states.

#### CMO #7: Youth appreciate how to use mental health knowledge to support friends and family

Youth participants described how their learning in Agenda Gap supported their ability to communicate and provide non-judgemental mental health support to their family and friends. They discovered how to be a better listener, how to avoid prematurely trying to solve others’ problems, and how to be open to diverse ideas and views.

*[Agenda Gap] helped me better help other people because mental health promotion focuses on mental wellness…[now] I focus less on fixing things and more on empathizing*. *[6Y*, *610]*

Youth also felt empowered to challenge regressive ideas around racism and mental health in peer groups and in family relations. Role modelling by Agenda Gap facilitators created “ripple effects”–or impacts extending beyond direct programme participants–in which youth became role models for positive, strengths-based approaches to mental health within their broader social networks.

*I learned so much about racism…and like how it affects someone’s mental health*. *I wouldn’t have been as comfortable to stand up for myself and my point of view…but I did it*. *And that was because of Agenda Gap*. *[3Y*, *67]*

*Context*. Having conversations about mental health with friends and family can be difficult due to stigma about mental illness and societal norms that silence such discussions.

*Mechanism*. Agenda Gap offered youth opportunities (resource) to explore different ways of engaging in conversations about mental health in their everyday lives. Youth gained confidence to speak about mental health (response).

*Outcome*. Improved ability to have sensitive and informed mental health discussions with family and friends; enhanced capacity to support family and friends with their mental health needs.

### Section C: Youth become inspired and informed to act as advocates and policy change agents

Agenda Gap mechanisms led to youth feeling motivated and equipped to take formal actions to effect policy change. This motivation arose from the intergenerational bond established with facilitators and feeling heard by adult allies who provided support and guidance to youth participants in relation to their policy goals. Many of these adult allies encouraged youth to voice their ideas. In the process of becoming policy change agents, youth learned to become more refined and strategic in their actions when they observed injustices. These efforts were made possible by the enhanced support system they acquired through Agenda Gap. Section C describes how youth grew their competencies and motivation for effecting systems change to promote and protect mental health among their peers and broader communities.

#### CMO #8: Youth identify policy gaps and initiate actions intended to create systems-level change

An important aspect of the work of the facilitators was to encourage youth to imagine possibilities and feel inspired to contribute to creating systems change. Through these realizations, youth were able to identify gaps in policies in their school and community environments that affect youth mental health and to work to effect change. These learnings became tools that helped youth to continue to identify gaps, harms, and inequities, leading to calls to action and seeking out adult allies to support these initiatives.

*I look out for gaps in the agenda more often now*. *I’ll see places where it doesn’t work and where the agenda is not focused on youth*, *even though it’s a youth centric environment… [for example] there’s a policy in the…teachers’ professional contract that you can’t allow your students to speak negatively about other teachers*. *And I said*, *“okay*, *I have never seen a teacher in my school stand up against bullying before*.*” I remember a time when I was being terrorized by 30 people and nobody said anything*, *that’s an agenda gap*. *I brought it up to my philosophy teacher and he said*, *“yeah*, *I will commit right now to always standing up*.*” I was like*, *okay*, *change*. *[6Y*, *685]*

*Context*. Policies affecting youth may have unintended mental health consequences. These consequences may persist, in part, because youth are absent from policy discussions and have less power and influence in systems compared to adults.

*Mechanism*. Facilitators provide information on social determinants, equity, and youth rights (resource), helping youth to recognize when policies are unfair, inequitable, or harmful (response). Youth become empowered to speak up on issues affecting them and their communities (response).

*Outcome*. Youth voice concerns over gaps in policy; youth improve the policy environment to better meet youth mental health needs.

#### CMO #9: Youth become skilled in different approaches to advocacy and incorporate thoughtful, balanced responses to support action

Agenda Gap provided youth with perspective on the reality of advocacy and systems change. Youth gain insights into the nature of advocacy work and the extended time often required to effect change. Adult allies invited to support the programme highlighted the importance of being open and transparent with youth and helping them to create realistic goals. Youth described the confidence they gained through the support of adult facilitators and adult allies, even when immediate system change was not apparent. Youth increased their confidence in approaching school staff about mental health issues and gained a realistic sense of how adults may react. Youth expressed how they learned to balance or regulate their response in the moment. When they felt something was not right, they learned that there are alternative ways to effect change, such as speaking with an adult ally outside of the immediacy of a problematic event.

*…like school principals and teachers who had humiliated their students* …*who would like show a triggering film in class*. *And I’d be like*, *“Hey*, *my classmate had anxiety attacks because of this*. *Could you give a trigger warning?” And they’d be like*, *“I’m just doing my job*. *Don’t tell me what to do” …I think the higher up you go…maybe the less people fear losing what they have*, *and also going to people who have the time in their schedules and the resources and the specialty to help me out*. *So rather than going to random school teachers and being like*, *“Hey*, *maybe do this better” [it’s]… going to a school trustee who specializes in youth mental health*. *[6Y*, *478]*

*Context*. Youth who try to effect change in their school environments may be confronted with adults who are not moved to act on youth requests. Adult staff working in school systems may not always receive empowered youth voices with a sense of gratitude and value.

*Mechanism*. Agenda Gap provided youth with insights into the multiple channels available for effecting change (resource). Through this process, youth gained confidence to make suggestions to adults, including in the school system and beyond (response).

*Outcome*. Youth have a more refined, balanced approach to addressing problematic events and committing to a longer-term view of change.

#### CMO #10: Youth gain awareness of adult ally perspectives on youth mental health promotion and experience expanded networks and strengthened relationships with non-familial adults

Youth described how gaining adult allies through Agenda Gap helped them to realize how adults working in mental health and policy perceive youth mental health needs. Youth were exposed to adult allyship, which some expressed as a shocking contrast to their everyday experiences with adults. These programme experiences helped change views on intergenerational linkages and the support adults can bring to effect change.

*One of the adult [allies] in that group said something about how “youth are the experts and they need to be heard first and foremost and then the rest of [the adults]…should just try their best to make their ideas come to life”…It was just very shocking*, *in the best way possible*. *[15Y*, *540]*

By changing their perceptions of adults and gaining an appreciation that adults can be allies, youth expressed how Agenda Gap helped to build linkages with adults working in key areas supporting youth mental health. Youth said that they felt motivated and empowered to reach out to these allies in times of need and learned to collaborate with them to create change.

*Before [Agenda Gap] I kind of saw it [as] youth versus authority*, *and now it’s like youth with some authorities*. *Maybe others won’t be so supportive*, *but we have supports now*. *It’s restored my faith in adult allies*, *and I’d be more willing to reach out to adults in the future about needing help with an initiative*. *[6Y*, *478]*

Youth further explained how the positive experiences they had with adult allies gave a sense of optimism that they could communicate and forge links with other adults in their everyday lives, including those who are not specifically youth mental health policy makers.

*If Agenda Gap didn’t invite her [adult ally] and…if she didn’t speak about racism and how she faces it…I wouldn’t have felt comfortable to ever email her*. *But now*, *if there’s a problem at my school*, *I could email her easily because I know that she’s nice*, *she’s friendly*. *She wants students to have their voices heard and stuff like that*. *[3Y*, *399]*

*Context*. Youth are typically unaware of how adults perceive youth mental health and how adult stakeholders working in youth mental health and policy perceive their needs.

*Mechanism*. Agenda Gap provided exposure to adult decision maker perspectives through the inclusion of adult allies (resource) allowing youth to become aware of the adult perspective on youth mental health needs and how this may contrast with their perspective (response).

*Outcome*. Youth fostered intergenerational connections with adult allies; explored new forms of allyship with adults in their everyday lives.

## Discussion

Mental health promotion provides a promising, strengths-based orientation to creating the conditions for good mental health and addressing issues of equity for youth and communities. Yet while there is growing evidence illustrating the benefits of mental health promotion programming in various settings, there remains a paucity of literature examining the role of non-familial youth-adult relationships in the context of mental health promotion programming for young people. Drawing on realist methodology, this study addresses this knowledge gap and articulates key causal mechanisms of impactful youth-adult relationships using the Agenda Gap programme as an exemplar. These findings contribute important data-driven and theory-supported insights that are critical to advancing the mental health promotion field and related outcomes for youth and their communities.

While the broad early impacts of Agenda Gap have been detailed elsewhere [5], this paper provides additional insights into the outcomes of the programme. Aligned with our past mixed methods findings, the present analysis shows programme outcomes across individual, interpersonal, and community levels [5] At the individual level, our realist evaluation data show that Agenda Gap contributed to improved mental health, coping skills, and confidence among participants alongside sustained interest and engagement. At the interpersonal level, outcomes centered on increased sense of trust and healthy youth-adult relationships, as well as new capacity among participants to support the mental health of friends and family. At the community level, Agenda Gap outcomes included youth-driven advocacy and community change efforts as well as new understandings about how adults can share power and create conditions for positive youth engagement. These outcomes are also congruent with findings from Mathias and colleagues (2019) who conducted a realist evaluation of the Nae Disha mental health promotion programme for youth with mental health challenges in northern India [35]. These authors reported similar multi-level outcomes focused on improved personal skills, capacities, and mental health, alongside enhanced relationships (in this case, peer), and social inclusion. This alignment in outcomes across diverse programmes and delivery contexts warrants further exploration to extrapolate the mental health promotion programme features, or mechanisms, that lead to these shared positive outcomes.

Beyond the outcomes, the present analysis narrows in to illustrate how youth mental health–and associated skills and competencies–can be cultivated through mechanisms linked to the development of trusting non-familial youth-adult relationships in a group-based mental health promotion setting. Overall, Agenda Gap youth participants reported improved personal mental health, enhanced confidence in discussing mental health with friends and family, and the development of new advocacy skills that can be applied to influence mental health promoting changes in school and community systems. Moreover, youth identified individually and collectively benefiting from expanded and more nuanced understandings of mental health. The recognition and application of distinctions between positive mental health and mental ill-health, as has been articulated by others [36], provided opportunities to surface community assets and inform policy-level solutions aimed at enhancing mental health among youth and their wider communities.

This strengthened capacity for youth to envision and contribute to policy-level solutions evokes consideration of the ways that structure and agency operate within the context of a youth mental health promotion programme such as Agenda Gap. Structure, which is operationalized and sustained through policy decisions (rules), shapes and constrains the life opportunities afforded to different people and groups and can contribute to the production and re-production of inequities, including those related to mental health and illness [37]. Agency, on the other hand, is reflected in “capacities to initiate, perform and maintain actions in order to achieve socially mediated outcomes…[and] can also be reflected in the experiences of making choices or decisions” [38], p.4. This concept can be further delineated to acknowledge individual and collective forms of agency. The latter is associated with group settings that support relational processes conducive to catalyzing transformative, structural-level change [38],–a CMO that was particularly salient in the Agenda Gap interview data. Indeed, Agenda Gap provided youth with an intergenerational, relational space that was intentionally designed to support autonomy building. This included practices of ongoing reflection, and opportunities for youth to make informed choices and collectively identify the social and structural factors affecting young peoples’ well-being. Sharing these experiences in a safe and supportive setting created the required conditions for Agenda Gap youth to contribute to addressing structural inequities through collective agency that could be leveraged, regardless of social positioning, to instigate transformative change.

While these benefits were prominent among youth participants, Lorimer, Knight and Shoveller [38] note that impactful youth-led structural change often requires adult allies who can contribute various resources. This was evident in the Agenda Gap interview data, in that adult allies gained and communicated a deep respect for the important contributions that youth can offer to policies impacting mental health. This illustrates how the causal mechanisms of non-familial youth-adult relationships may be activated not only among youth, but also among affiliated adults in positions of power. Developing empirical measures to test how the relational qualities of these intergenerational relationships operate to build collective agency and resultant structural transformation will be an important development for the mental health promotion field.

Grounded in the patterns derived through analysis of the Agenda Gap interviews, the elements underpinning the impactful nature of the intergenerational relationships between youth participants and adult facilitators were further distilled. A critical mechanism in the approach to programme facilitation was described by youth as ‘guiding without directing.’ This relational practice, widely understood as critical to healthy and productive youth-adult relationships [39] figures prominently in the theories of Third Space and Third Place. Here, co-created environments outside of family and school settings provide new–or unfamiliar–opportunities for intergenerational connection [31, 40]. Linking these theoretical ideas to the dataset revealed how Agenda Gap exemplifies a counterspace. This counterspace is a particular form of Third Place that promotes adaptive responding–or the capacity to achieve and sustain well-being despite contextual constraints [33]. This allowed for direct relational transactions where strengths-based narratives from and about young people could be actively constructed and affirm positive youth identities [35]. Guiding without directing meant that adult facilitators maintained an equipoise toward youth voices, engaged in informative, albeit non-directive rapport building. This constructed an atmosphere of humility and respect for diverse opinions and expressions. Adult facilitators actively worked to minimize relational distance without losing their own identities. This supported them in retaining the ability to provide protections, guidance, and role modelling as appropriate, resulting in a safe, informal, and social atmosphere characteristic of a Third Place. These practices further fostered genuine connection and mutual learning, set apart from paternalistic intergenerational hierarchy on the one hand, and casual peer-like intergenerational friendship on the other. Leveling this social power hierarchy afforded youth participants the freedom to express their individual and collective perspectives and build a strengths-based group identity to enact personal and community change.

While this paper makes an important contribution to understanding several of the key causal mechanisms underpinning impactful mental health promotion programming for youth, there are limitations that warrant consideration. Namely, the data utilized in this paper reflects the perspectives and experiences of youth and adult ally participants from a single Canadian province. Though these participants bring diversity in lived experiences and socio-cultural identities, they predominantly identify as women/girls and other aspects of their identities (e.g., sexual orientation) were not explored. Additionally, while Agenda Gap was initially designed for in-person delivery, COVID-19 public health restrictions pushed early offerings to a virtual setting, which may hold implications for the nature or processes of relationship development. Within this context, it was encouraging that non-familial youth-adult relationships featured so strongly in the data on programme mechanisms of impact. Moreover, certain aspects of the virtual environment, such as the ability to remain anonymous while still contributing to group discussions, appeared as a strength of this delivery modality. There remains a need for further research reflecting other aspects of participant diversity and social context as well as delivery setting (i.e., in-person and hybrid) to strengthen and tailor conclusions.

## Conclusion

Addressing youth mental health through a comprehensive approach, inclusive of mental health promotion, is a global public health priority. Non-familial youth-adult relationships can contribute key causal mechanisms supporting the realization of mental health promotion programming goals. Through building a sense of collective agency and creating relationally embedded opportunities for youth to explore their ideas and lead systems transformation, adult facilitators of youth mental health promotion programming can create Third Space and Third Place counterspaces. These counterspaces can allow youth to build adaptive responses and catalyze their efforts to effect change in their personal spheres, informal relations, and wider communities.

## Supporting information

S1 Appendix Definition of terms for realist evaluation. (DOCX)

S2 Appendix Agenda Gap realist interview guide. (DOCX)
