# Supplementary material for: Identifying mechanisms of youth mental health promotion: A realist evaluation of the Agenda Gap programme
Source: PLOS Ment Health. 2024 Jun 18;1(1):e0000068. doi: 10.1371/journal.pmen.0000068 (PMC12798558; doi:10.1371/journal.pmen.0000068)
Supplement: S1 Appendix — (DOCX) [file pmen.0000068.s001.docx]

**S1 Appendix**

**Definition of Terms for Realist Evaluation**

| Context-Mechanism-Outcome Configurations: The central heuristic used in realist evaluation to understand what works, for whom, under which circumstances and how. Context is the external backdrop of programmes whereas mechanism is how stakeholders respond to resources inside the boundaries of a program. Outcome is measurable impact at the behavioural, clinical or system level.  For example:  *Context: youth often experience a discrepancy between their experiences of mental health and the communication about mental health with adults in their lives (parents, teachers, etc.)  Mechanism: adult facilitators of the Agenda Gap programme offer a non-judgemental, caring space for youth to express their ideas about mental health (mechanism resource) leading youth to feel comfortable exploring ideas and gaining new realizations (mechanism response).  Outcome: Increased youth investment and participation in the programme; new forms of youth-centred mental health support, advocacy and policy.*  Initial Programme Theory: A hypothetical statement, often in the form of ‘if…then,’ developed at the start of a realist evaluation to explain how a programme or programme component works to produce outcomes. IPTs can take the form of rough, non-specified CMO configurations. For example: *“If young people interact with policy makers who are unresponsive or pessimistic in relation to youths’ ideas for change, youth may feel frustrated and disillusioned, resulting in abandoning change efforts. However, if youth receive emotional support from Agenda Gap facilitators, despite discouragements, they may then persist in their change efforts and build resiliency for advocacy work now and in the future.”*  Middle-Range Theory (MRT): Conceptual theory that can be used to scaffold programme theories and provide a lens on data for ontologically-deep analysis [1]. MRT is not abstract to the point of being disconnected from the on-the-ground workings of programmes, yet not so specific to pertain to one programme exclusively [2].  Programme Architecture: The complete set of strategies/components that comprise an intervention, both formally allocated and advised, as well as informally assembled and adapted from local resources and deficits [3].  For example:  Youth-relevant policy: Youth relevant policy is policy that influences the conditions in which youth grow and develop. In Agenda Gap, the policy focus is collectively identified by youth participants and influences the conditions required for positive mental health and well-being (e.g., appropriate and inclusive school policies that create a sense of safety, health policies that support intervention addressing the social determinants of mental health). |
| --- |

1. Jagosh J. Retroductive theorizing in Pawson and Tilley’s applied scientific realism. Journal of Critical Realism. 2020 Mar 14;19(2):121–30. Available from: https://www.tandfonline.com/doi/full/10.1080/14767430.2020.1723301

2. Merton RK. On theoretical sociology: five essays, old and new. New York, NY: Free Press; 1968.

3. Jagosh J, Stott H, Halls S, Thomas R, Liddiard C, Cupples M, et al. Benefits of realist evaluation for rapidly changing health service delivery. BMJ Open. 2022 Jul;12(7):e060347. Available from: https://bmjopen.bmj.com/lookup/doi/10.1136/bmjopen-2021-060347
