# Supplementary material for: Identifying mechanisms of youth mental health promotion: A realist evaluation of the Agenda Gap programme
Source: PLOS Ment Health. 2024 Jun 18;1(1):e0000068. doi: 10.1371/journal.pmen.0000068 (PMC12798558; doi:10.1371/journal.pmen.0000068)
Supplement: S2 Appendix — (DOCX) [file pmen.0000068.s002.docx]

**S2 Appendix**

**Agenda Gap Realist Interview Guide**

1. I am hoping to understand a bit more about your experiences in the programme and I am wondering if you could tell me about the most important aspects of your involvement with the Agenda Gap (Seeking information on anything they feel important to share about their involvement. Mostly an opportunity to break the ice and create dialogue).
2. **(Selection)** Before you started with the Agenda Gap, you participated in a one-on-one interview with one of our research team members and had some discussions about the role. Can you tell me about how that experience influenced your decision to participate?

- How did this interview process prepare you for your participation with the Agenda Gap? (Understanding role).
- Based on our experiences doing these individual interviews, we think that they support us in bringing together a group with shared experiences and interests and that this supports the identification of common policy goals as well as a safe space to share. What are your thoughts on this? Are there any other thoughts that you have about this process?

1. **(Incentivization)** Some research shows that providing payment to young people for their involvement in research or other programming has some effect on their participation. Based on your own experience, do you relate to this in any way? What about your peers?

- It might seem simple, but could you tell us in more detail what payment means to you?
- If you were not paid, how would your participation have looked different?
- What, in your opinion, other things besides money would stimulate you, create and hold interest in being involved in the programme?
- in the Agenda Gap programme? (either things already in place like a certificate, being housed at UBC, or not yet included)

1. **(Direct impacts)** We expect that the Agenda Gap programme will improve policies for youth mental health. That’s one outcome, but we are also interested in the immediate positive or negative effects of being involved. Can you describe any positive impacts to you personally? Can you describe any negative impacts to you personally? (safety, self-efficacy, mental health, group cohesion, feeling valued)
2. (**ripple effects)** Now that you have participated in Agenda Gap, in what ways have you seen things shift (outlook, relationships) among your peers because of your participation?

- What shifts have you noticed in your school, at home or in your community?
- The activities that you and the group engaged in throughout the Agenda Gap programme were meant to support a shared policy change target and actions to improve your mental health as well as that of your community.) Probe for more detail around what is named – how was it accomplished or how could be improved if negative

1. **(Adult facilitator)** Can you talk a bit about your experience with the adult facilitators of the Agenda Gap programme? What impacts did your relationships with these adults have on you?

- Do you think the facilitators had an impact on your mental health and well-being? Others in the group?
- What worked for you? What is important for us to understand about the adult facilitator?

1. **(Facilitator – Allies)** This question is about the community partners and decision makers the Agenda Gap connected you and the group to. What would be important for us to know about those experiences? (Probe for: feeling supported, valued, heard and empowered)

- What did you hope would result from meeting with these partners?
- Did anything surprise you from your meeting with these partners?
- How could this process be improved? (probe for timing)
- Did having the opportunity to connect with these partners and decision makers impact how you would seek to connect with other adults/decisionmakers in the future? Tell us about this.

1. **(Facilitator managing expectations)** Policy change can take a long time. Did the facilitators prepare you for that and if so how? If not, what would you recommend facilitators do to prepare people?

- Did noting the group’s successes along the way by the facilitators impact your experience of the policy change process?

1. **(Facilitator - safety)** The group built a community safety agreement. Tell us a little about that. Were there any moments or experiences you or others had where it didn’t feel safe to share something? How did this impact your experience in the programme? Are there other strategies that we could incorporate to build a safe environment in the future? (probing for managing conflict, trust, confidence).
2. **(Teaching & learning content)** The Agenda Gap programme included content about mental health promotion, youth rights and intersectionality. How did this content influence your understandings of mental health? Did it affect your choice of policy goals? In what ways?

- How did this content change (or strengthen?) your thoughts on what should be done to support youth mental health?
- Was there anything that particularly stood out from the training that impacted your understandings of mental health?
- How did the opportunities to apply these understandings/approaches to mental health shape your own mental health? What about within your broader community, have any of these ideas you were learning about get shared with others? Tell us about that.

1. **(Teaching & learning strategies)** Agenda Gap sessions included a variety of activities and different ways of learning and engaging. What was your experience of the programme variety? How important do you think this variety is and why? (Probe for: critical thinking, knowledge gains, interest, retention and motivation of the participants)

- What were your favorite topics?
- What were your greatest learnings?
- What could be improved?

1. **(Program design)** Agenda Gap was designed with different phases. The first was intended to provide a solid grounding in knowledge that could be helpful in policy advocacy efforts. The next phases were left intentionally open-ended to allow for the focus to emerge from the group as a way to foster youth ownership of the process and support sustainability of advocacy efforts beyond the conclusion of the formal programme. How was your experience of this programme design?

- What do you imagine would be helpful in supporting groups of youth to truly take the reins and direct the later activities of the group?

1. **(SEL)** In addition to the variety of activities included, the Agenda Gap sessions had skill building activities to help for handling emotions and coping with stress. Did these activities support you? If yes, how did they do that?

- What would you recommend we include in future sessions?

1. **(Group format)** The Agenda Gap programme is delivered in a group format. Could you tell us a bit about your experience of the group process? How did the group meet your needs, or not? (probing for: social connectedness, enhanced sense of agency, improved mental health literacy, and identification of collective foci for action).
2. **(Timing/flexibility)** Can you talk about your experience of the time commitment as well as the pacing of material? Were there any barriers to your participation? If so, how did the facilitators respond? Did it help? (Probing for: increased youth interest, engagement, and retention in the programme).

- How do you feel about the length of the sessions? (too long or too short?)
- Tell me about particular sessions that felt more engaging than others.

1. **(Youth centred)** Can you share a bit about your experience of being a youth collaborator? Do you feel you and the other youth helped guide the activities? Tell us more about that. How would your interest in participating have changed if the programme was not as youth driven? (probe for: enhancing retention, increasing participation and promoting mental health)
2. **(Future)** When we run Agenda Gap in the future with other youth, what is the most important thing we should do?

- What do you think would happen if we didn’t do this?
- Anything else that we should know?
